# Can native clonal moso bamboo encroach on adjacent natural forest without human intervention?

**DOI:** 10.1038/srep31504

**Published:** 2016-09-07

**Authors:** Shangbin Bai, Yixiang Wang, Richard T. Conant, Guomo Zhou, Yong Xu, Nan Wang, Feiyan Fang, Juan Chen

**Affiliations:** 1 Jiyang College, Zhejiang A & F University, Zhuji, Zhejiang 311800, PR China; 2 Department of Ecosystem Science and Sustainability, Colorado State University, Fort Collins, CO 80523, USA; 3 Zhejiang Provincial Key Laboratory of Carbon Cycling in Forest Ecosystems and Carbon Sequestration, Zhejiang A & F University, Linan, Zhejiang 311300, PR China

## Abstract

Native species are generally thought not to encroach on adjacent natural forest without human intervention. However, the phenomenon that native moso bamboo may encroach on surrounding natural forests by itself occurred in China. To certificate this encroaching process, we employed the transition front approach to monitor the native moso bamboo population dynamics in native Chinese fir and evergreen broadleaved forest bordering moso bamboo forest in Tianmu Mountain Nature Reserve during the period between 2005 and 2014. The results showed that the bamboo front moved toward the Chinese fir/evergreen broadleaved stand with the new bamboo produced yearly. Moso bamboo encroached at a rate of 1.28 m yr^−1^ in Chinese fir forest and 1.04 m yr^−1^ in evergreen broadleaved forest, and produced 533/437 new culms hm^−2^ yr^−1^ in the encroaching natural Chinese fir/evergreen broadleaved forest. Moso bamboo coverage was increasing while adjacent natural forest area decreasing continuously. These results indicate that native moso bamboo was encroaching adjacent natural forest gradually without human intervention. It should be considered to try to create a management regime that humans could selectively remove culms to decrease encroachment.

The invasion of exotic species has become a global problem and received increasing attention^1^,^2^. Invasive non-native plants can occupy the space and reduce the biodiversity of native ecosystem composed of local species, disrupt nutrient and hydrologic cycles, and modify the disturbance regimes and geomorphology of invaded ecosystems^3^,^4^. Understanding the mechanisms underlying biological invasions is crucial to evaluating invasions and will benefit for the management and restoration of invaded ecosystems^3^,^5^. However, almost all of the area expansions reported are about non-native species. The studies that quantify expansion or overabundance of native species are scarce. Some native species became overabundant in their natural distribution range that occupied the space of other native species like exotic species invasion, impacting seriously local ecosystem^6^. These negative effects are often paid less attention when the overabundant species is economically valuable.

Bamboo is one of most valuable plants providing with lots of goods and services. Its remarkable growth rate and versatile properties such as renewability, strength and the high number of applications, have made it one of the most important plants^7^. Bamboo forests are important for biodiversity, from providing food and shelter to large animals (e.g. Giant Pandas and Mountain Gorillas) and birds, to soil organisms, insects, and other plants and shrubs that together make up the bamboo forest ecosystems^8^. Moso bamboo (*Phyllostachys edulis*) is one of the most widespread subtropical bamboos in the world. It is mainly distributed across southern China, including 12 provinces such as Fujian, Jiangxi, Zhejiang, Hunan, etc. Moso bamboo is the fastest growing plant in the world that can grow up to 119 cm in one day and 24 m high in 40 to 50 days^9^. From March to May is the fast growth period for shoots and new culms, while from July to September is the period of rhizome growth and shoot bud division^9^. Moso bamboo was introduced to Japan in 1736 from China^10^. As an exotic species, moso bamboo forest invasion was found 30 years ago in Japan. Moso bamboo has invaded secondary deciduous broad-leaved forests in eastern Japan near Tokyo^11^,^12^ and in central Japan near Kyoto^13^, broadleaved forest, coniferous forest, bush and grassland near Hirasawa and Kofuki of eastern and western Japan^14^. Such phenomena revealed that the moso bamboo has potential invasiveness.

In China, the moso bamboo plantation area has been increasing steadily since 1990, not only for edible shoots but also for mature culms harvesting. Recently, moso bamboo plantations area gained faster development not only for the economic value but also as a significant carbon sink especially in Zhejiang province^15^. Moso bamboo forests cover about 3.37 million hm^2^, about 2% of the total Chinese forest area and about 70% of the total Chinese bamboo area^16^. The rapid development of moso bamboo plantations has assisted local economic situation on the one hand, but on the other hand they may bring about expansion into natural forests. Ding *et al*.^17^ reported that the area of moso bamboo forests increased dramatically at the speed of 4.47 hm^2^ yr^−1^ from 1985 to 2003 occupied the space of surrounding natural forests in Tianmu Mountain National Nature Reserve of Zhejiang Province by using remote sensing and ascribe to few human disturbance for the moso bamboo area expansion, but no information is about the encroachment process. Coincidentally, our initial view was that moso bamboo may expand into adjacent natural forest by itself according to our repeatedly field observation before 2005. Similar to exotic species, expanding native moso bamboo can reduce natural diversity by monopolizing resources, changing the species composition or relative abundance of sympatric species, and even altering forest structure^17^,^18^,^19^. However, whether native moso bamboo can expand into natural forest remains a point of considerable debate. One of the fundamental questions in this debate is whether moso bamoo expansion is dependent on human support or only by itself. In order to clarify the bamboo encroachment process, we started in 2005 to set up permanent plots to test the hypothesis that moso bamboo as a native species can expand into surrounding natural forest sites by itself.

The study of invasions of bamboo in Japan has primarily relied on retrospective methods which are inherently biased toward introduced earlier^12^. However, it is not clear whether the expansion of moso bamboo founded in Japan is a result of human activities or its exotic that made it become invasive. A key point to address it is whether native stands of moso bamboo are expanding their space in China by itself. Although some related research has reported that abandoned moso bamboo plantations are quite invasive in Japan^11^,^12^,^14^, the human activities cannot be excluded definitely. Expansion of bamboo using remotely sensed images collected at different times is unable to characterize pre-existing, human-driven factors that may have fostered invasion. Long-term field data documenting how moso bamboo expands into new habitats and impacts native forest communities over time are not available. Especially as a native species in China, whether moso bamboo can encroach on adjacent natural forest eliminating human support is still lack of information at individual and stand level based on long-term continuous observation. Given such limited research, uncertainly remains over the expansion propensity of moso bamboo.

The objectives for this study are as follows: (i) to test the hypothesis that moso bamboo can encroach into the adjacent forests without human activities with solid proof; (ii) to quantify encroachment rates, and iii) to determine whether there were different encroachment rates when expanding into different forest types by moso bamboo. We addressed these objectives by monitoring the location of new bamboo produced in the bamboo edge zone with two adjacent forest types (Chinese fir and evergreen broadleaved forest respectively) every year during the period 2005 to 2014.

## Materials and Methods

### Site descriptions

The study site is located in the Tianmu Mountain National Nature Reserve (119°23′47′′–119°28′27′′ E, 30°18′30′′–30°24′55′′ N), which belongs to typical bamboo distribution regions in Zhejiang province of southern China. According to forest resource inventories of the reserve, there was roughly 55.1 hm^2^ of moso bamboo forests at the time of reserve establishment. This coverage increased to 87.5 hm^2^ by 2004. There were no trees or bamboo planted by humans during this period due to nature reserve policies. Bamboo forests have also not been managed via any type of pruning or selective removal by humans, such that dead and falling bamboo culms can be frequently seen throughout the reserve. Before 1956, moso bamboo encroachment on adjacent local forests might have been limited by human harvesting shoot and culm in surrounding forest areas. It is illegal to harvest forest resources in this area since Nature Reserve established in 1956 with the approval of the Government.

Tianmu Mountain National Nature Reserve lies in the northern limit of mid-subtropical zone covering a total area of 4,284 hm^2^. The climate is damp monsoon climate with an annual precipitation of 1390–1870 mm and an annual temperature of 8.8–14.8 °C. The reserve is one of the sites with the richest subtropical higher plant species in China. There are 2,160 species of higher plants. Among them, more than 37 species are named after Tianmu Mountain and 1,200 species of medicinal plants. The vegetation type in this area is very rich, covering evergreen and deciduous broadleaved forest, bamboo forest, coniferous forests, marshes and aquatic vegetation under protection. The soils are red soil (<600 m), yellow soil (600–1200 m), and brownish yellow soil (>1200 m).

The moso bamboo population in this reserve is mainly distributed in the broad-leaved forest and Chinese fir forest with a distinct storey layer. The arbor layer is dominated by *Cyclobalanopsis glauca*, *Castanopsis sclerophylla*, *Schima superba*, *Cryptomeria fortunei* and *Cunninghamia lanceolata*. The shrub layer is dominated by *Camellia fraterna*, *Symplocos caudata*, *Rhododendron ovatum*, and *Lindera glauca*. The grass layer is dominated by the herbs of *Gramineae*, *Compositae*, *Cyperaceae* and *Dryopteridaceae*.

### Experimental design

**Figure 1:**
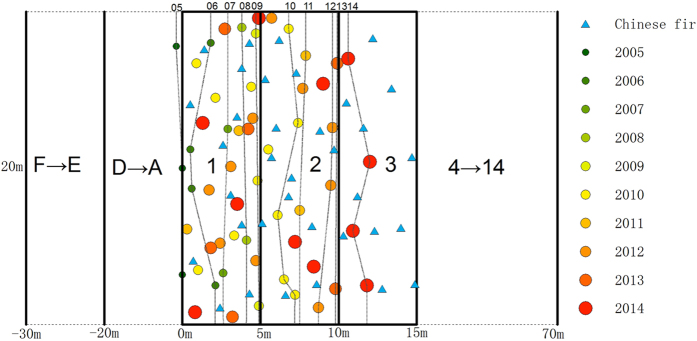
Dynamic distribution of moso bamboo culms in bordering Chinese fir forest during the period 2005 to 2014. All bamboo culms classified by birth year in subplot 1–3 were mapped. All Chinese fir stems in subplot 1–3 were also mapped. The 10 lines of 05 to 14 (abbreviation of 2005, 2006, ……, 2014) were the bamboo leading edge of each year. The pattern for evergreen broadleaved forest is not shown because it is very similar to the one for fir shown here. Note: F→E: Subplot E and F, mono moso bamboo stand; D→A: Subplot A, B, C and D, transistion zones; 1→3: Subplot 1, 2 and 3, Chinese fir stand without bamboo before 2005, presenting new shoots from 2005 to 2014; 4→14: Subplot 4, 5, 6,7, 8, 9, 10, 11, 12, 13 and 14, Chinese fir stand without bamboo shoots present before 2014. The encroachment path is from left to right.

To test the hypothesis that moso bamboo can encroach into adjacent forest, we selected two local typical forest types (i.e. Chinese fir/evergreen broadleaved forest) bordering native moso bamboo forest. In November of 2005, we selected six sites (three for each forest type) where are far from roads and tourist attraction and established one permanent plot in each site with a size of 100 m length × 20 m width along the encroachment pathway. It was comprised of three contiguous segments in order: mono moso bamboo, mixed transition areas, where moso bamboo mixed with Chinese fir/evergreen broadleaved, and the Chinese fir/evergreen broadleaved forest. Summary of moso bamboo, Chinese fir, and broadleaved stand characteristics see Table 1. The leading edge of the transition areas extends farther into the other forest type each year, with a well-defined line of moso bamboo in 2005. The sites are moderately sloped, approximately 15°, each site is at least 200 m apart. From this study was conducted, the plots were further protected to ensure nobody entering except this study needs. If bamboo forest can expand by itself, the leading edge will move forward. Therefore, this leading edge of 2005 was defined as a line beginning to expand in future years (Fig. 1). The plot was subdivided into 20 subplots of 5 m × 20 m. We defined the subplot No. 1 start from the leading edge toward Chinese fir/evergreen broadleaved forest. As Fig. 1 shows, the pure moso bamboo stand located in subplot E and F. Chinese fir showed in the other subplots except subplot E and F. Pure Chinese fir stand in 2005 located in subplot No. 1–14. Encroachment of bamboo was traced annually from 2006 to 2014. The locations of the arbor trees were measured by total station and mapped in each plot. For each new culm that emerged in front of the leading edge of 2005, we measured the location in the plot using total station, and recorded height, vitality (alive/dead), and the year in which emerged.

To get detailed information on bamboo rapid growth, total height of all bamboo shoots emerged in subplot 1–2 were measured weekly until height growth ceased for more than 2 weeks. The measurements took place between March and June in 2013. Heights were measured to the nearest centimeter with hypsometer, and diameters were measured with a tape with an accuracy of 0.1 cm. The vitality (alive/dead) of each tree and bamboo culm in each subplot was recorded in 2014.

### Expansion procedure mapping and expansion speed calculation

**Figure 2:**
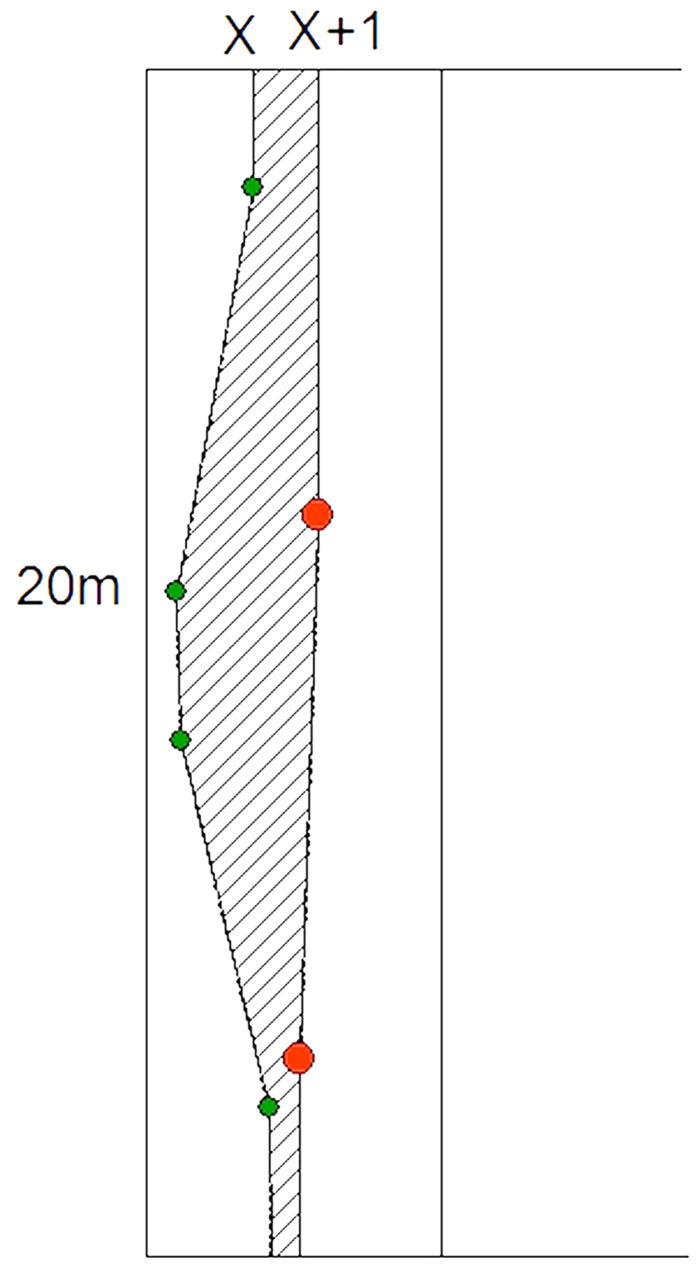
Calculation method of annual encroachment speed of moso bamboo. x + 1 line is current year’s leading edge and x line is the last year’s leading edge.

As shown in Fig. 2, if the bamboo forest leading edge moves toward the other forest type, the bamboo area will become larger and larger. The current expansion area was defined as the polygon difference between current year’s (x + 1) leading edge and last year’s (x) as the rest of boundaries fixed in the plot. The shade area is the expansion area at x + 1 year. The annual expansion speed could be determined by dividing the shade area by the width of plot (20 m for this study). If bamboo forest could not expand, the speed is 0, otherwise, it is bigger than 0.

### Statistical analyses

The data were analyzed using SPSS 18.0 software. Paired T test was used to determine the statistical significance of two forests on bamboo encroachment rate over the period. One-way ANOVA was performed to test the statistical significance of bamboo encroachment rate on the same forest among different years. Statistical significance for all analyses was set at *p* < 0.05.

## Results

### Dynamic encroachment process of moso bamboo

In 2005, tree stems were abundant in the non-bamboo forests and culms of moso bamboo were not present in subplot 1. From 2006 on, bamboo shoots began to appear. The bamboo front moved into the Chinese fir/evergreen broadleaved stand with new bamboo produced yearly. Therefore, bamboo occupied a broader and broader space that was dominated by Chinese fir/evergreen broadleaved trees before 2006, suggesting that moso bamboo was encroaching gradually. Figure 1 depicted the dynamic distribution of moso bamboo in adjacent nature Chinese fir forest during the period 2005 to 2014.

### Encroachment speed of moso bamboo

The encroachment speeds ranged from 0.57 m yr^−1^ to 1.80 m yr^−1^, with the average being 1.28 m yr^−1^ in Chinese fir forest and 1.04 m yr^−1^ in evergreen broadleaved forest (Table 2). There were significant differences between Chinese fir forest and evergreen broadleaved forest in the same year (*P* < 0.05). The coefficient of variation (CV) of the yearly speeds was 0.36 m yr^−1^ for Chinese fir stand and 0.38 m yr^−1^ for evergreen broadleaved forest. There were significant differences among different years in the same stand (*P* < 0.05). The mean rate of encroachment on Chinese fir forest and broadleaved forest by bamboo was approximately 1.65 m yr^−1^, 1.36 m yr^−1^ in rich years in which moso bamboo mainly bears lots of shoots and grow up into young bamboos, and 0.81 m yr^−1^, 0.64 m yr^−1^ in poor years in which moso bamboo mainly changes leaves and produces new rhizomes, respectively.

### Changes of culm density during the encroachment of moso bamboo

**Figure 3:**
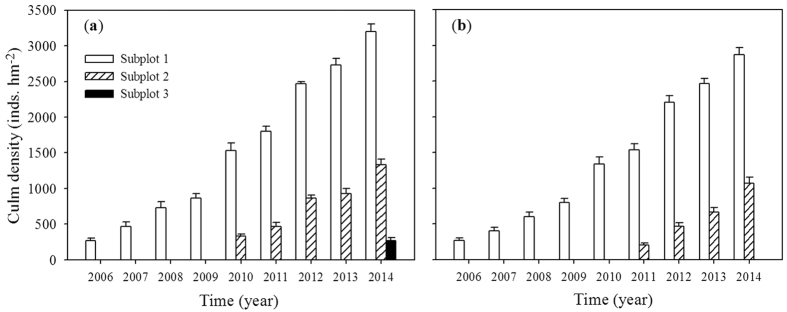
Dynamics of moso bamboo culms density in Chinese fir stand (a) and evergreen broadleaved stand (b) during the period 2006 to 2014. Subplot numbers are the same as in Fig. 1. Bars correspond to means and error bars are standard errors.

As Fig. 3 showed, bamboo density increased gradually not only in the adjacent Chinese fir forest but also in the evergreen broadleaved forest during the period. The moso bamboo produced 533/437 new culms hm^−2^ yr^−1^ in the encroaching natural Chinese fir/evergreen broadleaved forest. In subplot 1 the bamboo number increased from 0 inds. hm^−2^ in 2005 to 3200 inds. hm^−2^ in 2014 in Chinese fir forest, from 0 inds. hm^−2^ in 2005 to 2866.7 inds. hm^−2^ in 2014 in evergreen broadleaved forest, respectively. The density of bamboo in subplot 1 was higher and decreaed in the expanding direction of subplot 2, where the bamboo began to occur in 2010 for Chinese fir forest and in 2011 for evergreen broadleaved forest. By contrast, the bamboo density increased gradually from 0 to 1333.4 inds. hm^−2^ in subplot 2 and 266.7 inds. hm^−2^ in subplot 3 of Chinese fir forest, and 1066.7 inds. hm^−2^ in subplot 2 of evergreen forest in 2014. It meant there was no bamboo in bordering natural forest in 2005. But more and more bamboo appeared in the adjacent forest and moved farther and farther during the period 2006 to 2014, the mixed bamboo forest area becoming larger and larger. Although dead culms of moso bamboo were found in subplot 1, the bamboo forest may regenerae new culms yearly and increase density autonomously.

## Discussion

### Encroachment evidence of native moso bamboo

We proved that the moso bamboo front expanded automatically in both natural Chinese fir forest and evergreen broadleaved forest without human support by ten years permanent plots observation. This expansion is similar to the results from nonnative areas where moso bamboo has been introduced from China^13^. Okutomi *et al*.^12^ reported that the area covered by exotic moso bamboo forest had invaded by a factor of 2.7 over last 26 years (1961–1987) in southwestern parts of Tokyo in Japan. Additionally, Ding *et al*.^17^ also used remote photographs to document bamboo expansion rate of 4.47 hm^2^ yr^−1^ by detecting the bamboo area changes between 1985 and 2003 in Tianmu Mountain Nature Reserve in China. But some researchers denied the bamboo expansion naturally as a native species and argued that bamboo encroachment into forest took place must be in the case of requires human management^14^. Based on above considerations, this study was conducted in the sites where eliminated human support and the results of 10 years observation indicated that moso bamboo could encroach on adjacent forests by itself. Coincidentally, this study is similar to the expansion of native woody bamboos (*Guadua tagoara*) occurred in the Brazilian Atlantic Forest^6^.

In this study, we found moso bamboo could encroach into natural undisturbed Chinese fir forest and evergreen broadleaved forest and there were significant difference in the encroachment rate between them (Table 2), indicating that bamboo encroachment rate depended on adjacent forest types. The difference in expansion rate between Japan and China indicated that bamboo encroachment may be related to many factors, such as the bamboo itself, the soil type, the climate, the neighbor vegetation, and the topographical conditions of the expanded area.

### Mechanism of moso bamboo encroachment

**Figure 4:**
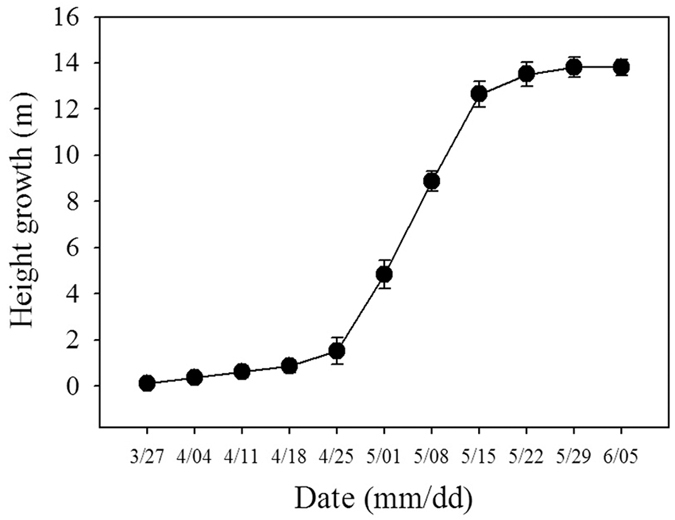
The rapid height growth of moso bamboo in subplot 1–2 of Chinese fir forest in 2013. Symbols correspond to means and bars are standard errors.

Moso bamboo encroaching on the adjacent forest mainly includes underground and aboveground processes. First, the underground rhizomes spread into neighboring forest, which cannot be seen by people. Then several years later bamboo sprouts appear from the underground rhizomes. Once the sprouts emerge from the earth, it will grow into bamboo in short time (only about 60 days) to finish stem elongation, reaching 12–15 m (Fig. 4). These characters allow bamboo to occupy the upper crown quickly. Moreover, bamboo shoots are highly shade tolerant. There are almost no leaves and branches when bamboo shoots initiate stem elongation. Maternal bamboo provides almost all of the energy and nutrients instead of its photosynthesis. Bamboo shade tolerance is manifest as low photosynthetic rates and rapid early growth. Most invasive species are not shade tolerant, so it is difficult for them to invade into forests with intact canopies or undisturbed communities^20^. However, some shade tolerant tree species, such as the Norway maple can invade intact forests and thus demonstrate the weak resistance to shade tolerant species in some forests^21^. The shade tolerance of bamboo shoots is beneficial for them to grow well beneath the dense canopy^22^.

We found that the bamboo encroachment speed differed each two years. This finding is consistent with a pattern of rich and poor year. Bamboo has the rich years bearing lots of shoots and growing up into young bamboos, and the poor year changing leaves and producing new rhizomes^23^. In this way, moso bamboo can encroach into adjacent forests intermittently quickly or slowly two-year cycle. This is different from Norway maple, which invades through seed dispersal and seed propagation at low frequency and long distance^21^. Although there are probably many factors that contribute to moso bamboo encroachment, we can affirm that this continuous encroachment of bamboo was inherent in local areas. In other words, undisturbed forests were not immune to the encroachment of moso bamboo.

Compared with the reserve after establishment, it can be guessed that the increase in moso bamboo area could also occur before the reserve was established, but local people actually harvested the moso and helped maintain it unconsciously to more manageable levels to prevent it from overrunning the other forests. However, the reserve establishment may cause or promote this increase due to nature reserve policies which prohibit people from harvesting bamboo culms and even bamboo shoots. In this sense, outlawing bamboo harvest inside the reserve was a detriment to this system.

### Implications of bamboo encroachment

Although moso bamboo is native in China, the proved ability of bamboo encroachment similar to exotic species invasion is somewhat alarming. Unconstrained moso bamboo encroachment could lead to the formation of the new bamboo forests, well-known in Eastern Asia^12^. The surrounding natural forest could be drastically disturbed. Such vegetation changes may alter forest floor microclimate with respect to light, temperature, and moisture^24^. Encroachment also potentially alters forest structure and dynamics and simplifies stand structure^18^, substantially reducing tree and shrub diversity in the forests^19^, modifying soil community structure, and increasing microbial biomass and taxonomic diversity^25^. The conversion of broadleaved forests to bamboo-dominated forests also may alter soil properties^26^ and reduced ecosystem storage of carbon (C) and nitrogen (N), having important impacts on regional C and N cycles^27^,^28^. In southern China, there are vast areas dominated by native moso bamboo (>3.37 million hm^2^)^16^. If rapid encroachment of moso bamboo forest into adjacent broadleaved forests continues in these subtropical areas, it would become ecological risk unexpected.

**Figure 5:**
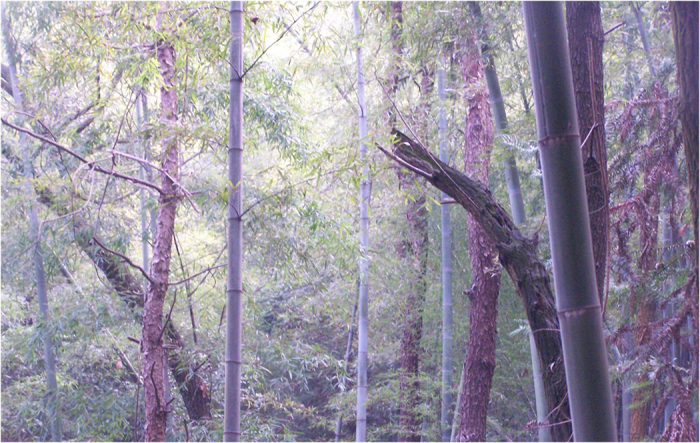
Moso bamboo encroaching on Chinese fir forest in subplot 2, forming the mixed forest of moso bamboo and Chinese fir at the Tianmu Mountain Natural Reserve. Some trees lost vitality and began to die just 5 years after moso bamboo encroaching (photo taken in 2013).

We found that even for protected undisturbed forests, it is difficult to resist the encroachment of bamboo. If this biological expansion is not to be controlled, moso bamboo seems likely to expand its area every year and decreasing the areas of evergreen broadleaved forest and Chinese fir forest. As shown in Fig. 5, the pure Chinese fir forest transformed to the mixed forest of moso bamboo and Chinese fir dominated by bamboo. Even some trees lost vitality and began to die one by one 5 years after moso bamboo encroaching. It warned us that the protected forests adjacent to moso bamboo in Tianmushan Nature reserve and other protected areas may be dominated by moso bamboo forest and losing their protecting value. These results have implications for the management and control of this encroaching species. Some actively strategies should be applied to decrease encroachment, such as digging a trench around bamboo stands or physically new shoots or culms each year.

## Additional Information

**How to cite this article**: Bai, S. *et al*. Can native clonal moso bamboo encroach on adjacent natural forest without human intervention? *Sci. Rep.* **6**, 31504; doi: 10.1038/srep31504 (2016).

## Figures and Tables

**Table 1:** Stand characters of moso bamboo, Chinese fir, and broadleaved forest (Means ± SE).

Stand type	Density (inds. hm^−2^)	Mean DBH (cm)	Mean height (m)	Canopy closure (%)
Moso bamboo forest	4210 ± 121	10.9 ± 0.4	12.1 ± 1.0	79 ± 4
Chinese fir forest	1200 ± 85	16.2 ± 0.5	13.2 ± 1.6	82 ± 6
Broadleaved forest	1465 ± 101	18.5 ± 0.8	14.7 ± 1.3	80 ± 5

**Table 2:** Encroachment speeds of moso bamboo from 2006 to 2014 (m yr^−1^)

Stand type	2006	2007	2008	2009	2010	2011	2012	2013	2014
Chinese fir forest	1.70 ± 0.23aA	0.78 ± 0.14aC	1.56 ± 0.27aB	0.84 ± 0.12aC	1.80 ± 0.26aA	0.81 ± 0.11aC	1.71 ± 0.28aA	0.80 ± 0.19aC	1.48 ± 0.27aB
Broadleaved forest	1.40 ± 0.17bA	0.57 ± 0.13bC	1.25 ± 0.24bB	0.67 ± 0.15bC	1.53 ± 0.25bA	0.62 ± 0.14bC	1.41 ± 0.20bA	0.68 ± 0.15bC	1.21 ± 0.21bB

Note: Data are means with standard error. Different lowercase letters in the same column indicate significant differences between stands in the same year (*p* < 0.05). Different capital letters in the same row indicate significant differences among years in the same stand (*p* < 0.05)
