# Erratum: Can native clonal moso bamboo encroach on adjacent natural forest without human intervention?

**DOI:** 10.1038/srep38269

**Published:** 2016-12-01

**Authors:** Shangbin Bai, Yixiang Wang, Richard T. Conant, Guomo Zhou, Yong Xu, Nan Wang, Feiyan Fang, Juan Chen

Scientific Reports 6: Article number: 3150410.1038/srep31504; published online: 09 07 2016; updated: 12 01 2016

This Article contains an error in the Discussion section.

“Some actively strategies should be applied to decrease encroachment, such as digging a trench around bamboo stands or physically new shoots or culms each year”.

should read:

“Some active strategies should be applied to decrease encroachment, such as digging a trench around bamboo stands or physically removing new shoots or culms each year”.

